# Development of a novel phantom for evaluating jawbone SPECT targeting medication-related osteonecrosis of the jaw

**DOI:** 10.1007/s11282-025-00809-2

**Published:** 2025-02-27

**Authors:** Naoya Hayashi, Norikazu Matsutomo, Ryotaro Tokorodani, Mitsuha Fukami, Miki Nishimori, Hitomi Iwasa, Kie Nakatani, Tetsuya Yamamoto, Takuji Yamagami, Tomoaki Yamamoto

**Affiliations:** 1 Division of Radiology, Department of Medical Technology, Kochi Medical School Hospital, Kohasu, Oko-Cho, Nankoku, Kochi 783-8505 Japan; 2 Department of Medical Radiological Technology, Faculty of Health Sciences, Kyorin University, B-524, 5-4-1 Shimorenjaku, Mitaka, Tokyo 181-8612 Japan; 3 Department of Diagnostic and Interventional Radiology, Kochi Medical School, Kochi University, Kohasu, Oko-Cho, Nankoku, Kochi 783-8505 Japan; 4 Department of Oral and Maxillofacial Surgery, Kochi Medical School, Kochi University, Kohasu, Oko-Cho, Nankoku, Kochi 783-8505 Japan

**Keywords:** MRONJ, SPECT, SUV, Phantom, Image reconstruction, Jawbone

## Abstract

**Objective:**

We developed a new phantom for technical evaluation of jawbone single-photon emission computed tomography (SPECT) examinations for medication-related osteonecrosis of the jaw (MRONJ). In this study, we verified the utility of the phantom by determining optimal image reconstruction parameters.

**Methods:**

We evaluated the image quality and quantification in jawbone SPECT images obtained by different reconstruction parameters using the phantom. The phantom images were acquired using a SPECT/computed tomography (CT) system and then reconstructed using ordered-subset expectation maximization (OSEM) iterative reconstruction with resolution recovery as well as scatter and attenuation correction with various update numbers and Gaussian filter full width at half maximums (FWHMs). The percent contrast (%contrast) and absolute recovery coefficient were calculated to determine the optimal reconstruction parameters (OSEM_jaw_). Nineteen patients with a clinical diagnosis of MRONJ who underwent bone SPECT/CT were enrolled for the clinical study. The performance of OSEM_jaw_ was verified by comparison with OSEM_current_ determined by a spherical phantom, using the correlation between the mean standardized uptake value (SUV_mean_) and clinical staging and visual assessment as endpoints.

**Results:**

In the phantom study, %contrast and absolute recovery coefficient increased with increasing update numbers. As the Gaussian filter FWHM increased, the quantitative accuracy and image sharpness decreased. The parameter determined by the phantom study (OSEM_jaw_) recommended 120 updates and no filter.

In the clinical study, the mean and standard deviation of SUV_mean_ obtained from OSEM_jaw_ were 8.9 ± 1.4 for stage 1 lesions, 12.9 ± 4.1 for stage 2 lesions, and 13.8 ± 1.4 for stage 3 lesions. For OSEM_current_, they were 5.4 ± 2.3 for stage 1 lesions, 8.3 ± 2.5 for stage 2 lesions, and 8.0 ± 0.9 for stage 3 lesions. The SUV_mean_ obtained from OSEM_jaw_ had a stronger correlation with clinical stage. Based on visual assessment, the quality of the SPECT images reconstructed by OSEM_jaw_ (3.7 ± 0.9) was superior to that reconstructed by OSEM_current_ (2.9 ± 1.1).

**Conclusions:**

We developed a novel phantom and adapted it for technical evaluation. This study demonstrated the utility of the developed phantom.

## Introduction

Medication-related osteonecrosis of the jaw (MRONJ) is a challenging condition arising during the administration of bone resorption inhibitors for the treatment of malignancies and osteoporosis, and its incidence has increased substantially in recent years. Assessing local bone inflammatory activity is important in the diagnosis of MRONJ and is performed by jawbone single-photon emission computed tomography (SPECT) imaging [1, 2]. Moreover, quantitative metrics, such as the standardized uptake value (SUV), are increasingly being used in MRONJ management [3–7]. Recent advancements in resolution enhancement, attenuation correction, and scatter correction have facilitated SUV-based quantitative evaluation in SPECT examinations, with numerous studies validating the utility of quantitative imaging [8–11]. Nevertheless, in nuclear medicine examinations using SPECT systems, quantitative accuracy is strongly affected by the radiopharmaceuticals’ radioactivity, acquisition parameters, image reconstruction parameters, and various correction conditions. Because data collection conditions vary among facilities, evaluating the appropriateness of each facility’s data collection protocols is crucial. In nuclear medicine, phantoms mimicking organs such as the brain, heart, spine, liver, and lymph nodes have been developed, aiding in the accumulation of evidence for technology and quantitative precision [12–22]. However, a phantom that reflects the characteristics of the jawbone has yet to be developed. Given its thin structure and unique shape, the jawbone is susceptible to partial volume effects, necessitating the establishment of suitable conditions to enhance image quality and quantitative precision. Therefore, the development of a new phantom could contribute greatly to increasing diagnostic accuracy and improving treatment outcomes in oral surgery.

In this study, we designed and developed a novel jawbone SPECT evaluation phantom based on human anatomy. We assessed the practicality and adaptability of the developed phantom by establishing appropriate parameters.

## Materials and methods

This study was approved by the ethics committee of our university (approval no. ERB-110089 and ERB-110327).

### Jawbone phantom

**Fig. 1:**
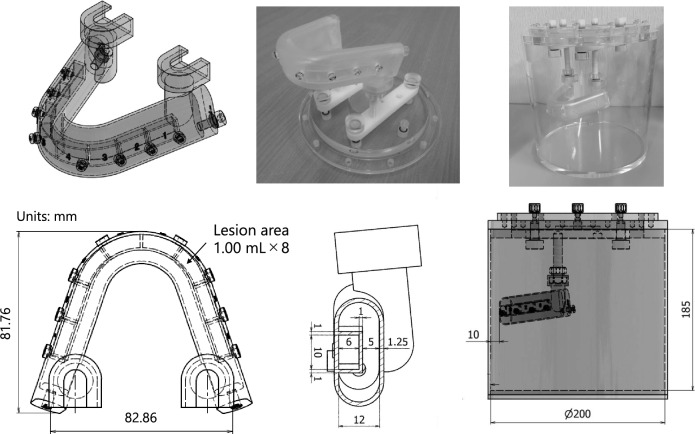
Photograph and design of the novel jawbone SPECT phantom. The phantom consists of a jawbone section and a cylindrical container. The jawbone section is a horseshoe-shaped structure, and eight lesion areas are set in the normal bone area

**Fig. 2:**
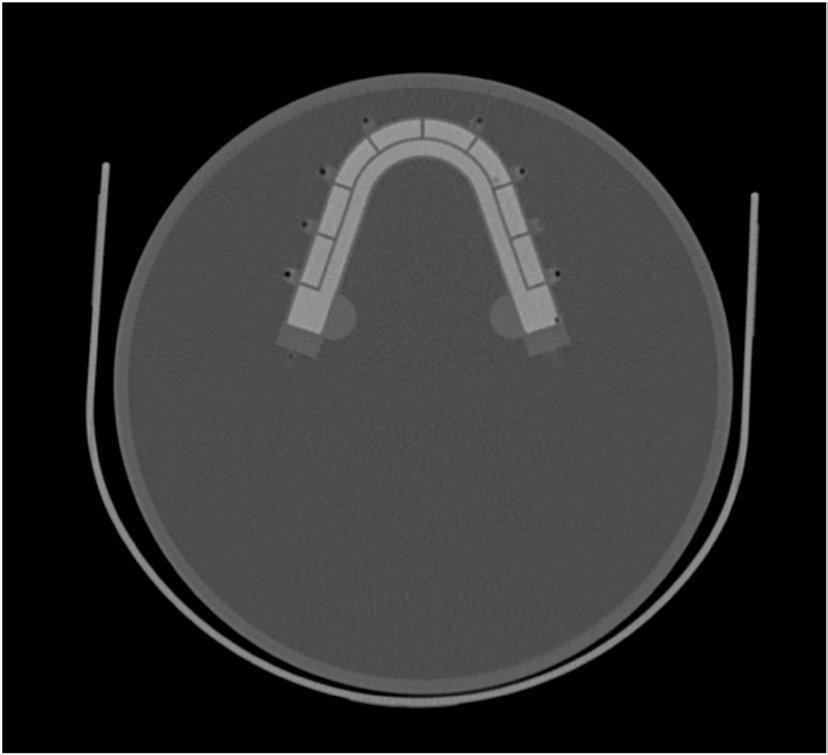
CT image of the novel phantom. The jawbone area was filled with a bone-equivalent solution (K_2_HPO_4_) with a concentration of 0.50 g/cm^3^, which results in a CT value comparable to that of the normal mandible

Figure 1 shows a photograph and the design of the novel jawbone SPECT phantom, which was designed based on 3-dimension (3D) skull data (https://free3d.com/ja/3d-model/skull-v3--785914.html). This phantom was created by outsourcing (NMP Business Support Co., Ltd., Hyogo, Japan). The phantom consisted of a jawbone section and a cylindrical container. The jawbone section was made of epoxy resin, forming a horseshoe-shaped structure 12 mm thick, measuring 120 mm front to back and 100 mm left to right. Eight lesion areas 1 mL in size were set in the normal bone area of 39.63 mL. Technical evaluation of jawbone SPECT could be performed by varying the radioactivity concentration, location, and size of the lesion. The cylindrical outer had a diameter of 200 mm and a height of 185 mm. It was designed to match the size of the Brain Tumor phantom, a ^11^C-methionine brain tumor PET phantom, considering the combined dimensions of the neck and maxillofacial. The outer container of the novel jawbone SPECT phantom was filled with 3.0 kBq/mL of ^99m^Tc solution, and the normal bone area was filled with ^99m^Tc in bone-equivalent solution (^99m^Tc: 39.0 kBq/mL and K_2_HPO_4_ solution: 0.50 g/cm^3^). K_2_HPO_4_ is a bone-equivalent solution with a composition comparable to that of cranium [23]. Figure 2 shows a CT image of the phantom. Simulated lesions of 1 mL were placed on both sides. The radioactivity ratios of the normal bone area to the lesions (lesion/normal ratio; LNR) were 1:2, 1:4, and 1:8 (lesion radioactivity: 78, 156, and 312 kBq/mL, respectively).

The concentrations of ^99m^Tc and K_2_HPO_4_ solutions were determined by comparing clinical and phantom evaluations in a preliminary study. The radioactivity concentration of the ^99m^Tc solution in the outer container was determined by placing a sufficiently large region of interests (ROIs) in the paracervical soft tissue and adopting the measured concentration. The concentration of the normal jawbone was determined by matching the measured radioactivity concentration of the normal side to that measured in the phantom. The measured radioactivity concentration ratio of the lesion to the normal area was 2.7 for the minimum, 4.2 for the median, and 7.9 for the maximum, so the LNR was set accordingly. The average CT value of the normal mandible was 530 Hounsfield units (H.U.), and the concentration of K_2_HPO_4_ solution was adjusted to obtain a similar CT value.

### Phantom study

#### Image acquisition and reconstruction

All SPECT data were acquired using a dual-head gamma camera (Symbia T6; Siemens AG, Erlangen, Germany) equipped with a low-energy high-resolution collimator. The system had a spatial resolution of 7.4 mm with ^99m^Tc placed 10 cm from the collimator. Image reconstruction was performed using the Syngo MI Apps version VA50C (Siemens Healthcare Co., Ltd., Munich, Germany).

The projection data were obtained using the continuous mode through 360° of rotation at 90 angular views. Data acquisition was performed with a 128 × 128 matrix, zoom of 1.23 × , pixel size of 3.9 mm, 360° acquisition (90 directions, 4° steps), and 25 s/step. A ^99m^Tc photo-peak window was set as a 20% energy window centered at 140 keV. A sub-window for scatter correction was set as a 7% energy window on the lower side of the photo-peak window. SPECT acquisition was performed in a circular orbit with a radius of 15 cm using an attachment for the brain. A low-dose CT scan was performed at 130 kV, with the quality reference set at 120 mA, auto exposure control for the tube current, rotation time of 0.6 s, slice thickness of 2.5 mm, and pitch value of 1.6.

The SPECT images were reconstructed by using the ordered-subset expectation maximization (OSEM) algorithm with resolution recovery, scatter correction, and attenuation correction, which used low-dose CT. The number of subsets was fixed at 10 and the number of iterations was varied from 1 to 15. The SPECT images were post-smoothed using a 3D Gaussian filter (no filter, full width at half maximum (FWHM) of 3.90, 5.85, and 7.80 mm).

#### Data analysis

To investigate the basic properties of the reconstruction parameters, percent contrast (%contrast) and absolute recovery coefficients (ARCs) were calculated. %contrast and ARC were used as evaluation indices to assess optimal image reconstruction parameters in terms of image quality and quantitative accuracy. We placed ROIs with a 5 mm diameter on the lesion and normal bone area on the CT images and then copied the ROIs on the SPECT images. The %contrast and ARC were calculated as follows:

1 $${\%contrast}= \left(\frac{Cl}{Cn}-1\right)/\left(\frac{Al}{An}-1\right)\times 100\left(\%\right)$$

2 $$\text{ARC}=\text{CCF}\times (Cl\text{ or }Cn)/(Al\text{ or }An)\times 100\left({\%}\right)$$

Here, *Cl* and *Cn* are the average counts in the ROI in the lesion and normal bone, respectively. *Al* and *An* are the radioactivity in the lesion and normal bone area, respectively. The cross-calibration factor (CCF) converts count values [counts/pixel] to radioactivity concentration [Bq/mL]. The CCF was obtained from the correlation between radioactivity and counts per second of a cylinder phantom. The cylindrical phantom had a diameter of 160 mm and a height of 150 mm. The CCFs were calculated and applied for each reconstruction parameter.

### Clinical study

#### Participants

We retrospectively enrolled 19 MRONJ patients who underwent bone SPECT examination at our hospital from April 2022 to February 2023. MRONJ diagnosis and clinical stage were confirmed according to the 2022 the American Association of Oral and Maxillofacial Surgeons definition [24]. The clinical stages are defined based on the presence of infection as determined by clinical findings. The relationship between stage and clinical symptoms is shown in Table 1. The patient characteristics are shown in Table 2. The patients included 7 men and 12 women with a mean age ± standard deviation of 72.0 ± 10.8 years (range: 41–86 years). The MRONJ stages of the patients were as follows: stage 1 (*n* = 6), stage 2 (*n* = 10), and stage 3 (*n* = 3). The patients’ target illnesses were as follows: cancer (*n* = 10), osteoporosis (*n* = 8), and rheumatoid arthritis (*n* = 1).

**Table 1:** The relationship between clinical stage and clinical symptoms

Stage 1	Exposed and necrotic bone, or fistulas that probe to bone, in patients who are asymptomatic and have no evidence of infection
Stage 2	Exposed and necrotic bone, or fistulas that probe to bone, with infection as evidenced by pain and erythema in the region of the exposed bone with or without purulent drainage
Stage 3	Exposed and necrotic bone or fistulae that probes tothe bone, with evidence of infection, and one or moreof the following: • Exposed necrotic bone extending beyond the region of alveolar bone (i.e., inferior border and ramus in the mandible, maxillary sinus, and zygoma in the maxilla) • Pathologic fracture. Extraoral fistula • Oral antral/oral-nasal communication • Osteolysis extending to the inferior border of the mandible or sinus floor

**Table 2:** Characteristics of all patients

Case	Sex	Age	Type of anti-resorptive agent	Target illness	Location	Stage
1	F	67	Zelodronate, denosumab	Breast cancer	Maxilla	2
2	M	83	Denosumab	Prostate cancer	Mandible	2
3	M	77	Denosumab	Prostate cancer	Mandible	1
4	F	68	Denosumab	Breast cancer	Maxilla	3
5	M	70	Denosumab	Lung cancer	Mandible	1
6	F	41	Denosumab	Breast cancer	Mandible	2
7	F	76	Ibandronate	Rheumatoid arthritis	Mandible	2
8	M	69	Denosumab	Prostate cancer	Mandible	3
9	M	60	Denosumab	Lung cancer	Mandible	2
10	F	66	Minodronate	Osteoporosis	Mandible	2
11	F	82	Ibandronate	Osteoporosis	Mandible	2
12	M	86	Zelodronate	Osteoporosis	Mandible	2
13	F	75	Denosumab	Osteoporosis	Mandible	3
14	F	81	Risedronate	Osteoporosis	Mandible	1
15	F	74	Ibandronate, minodronate	Osteoporosis	Mandible	2
16	M	75	Zelodronate	Lung cancer	Mandible	1
17	F	66	Denosumab	Osteoporosis	Mandible	1
18	F	87	Minodronate, denosumab	Osteoporosis	Mandible	1
19	F	63	Ibandronate, alendronate, denosumab	cervical cancer	Maxilla	2

*M* male and *F* female

#### Bone scintigraphy protocol

In all patients, ^99m^Tc-hydroxymethylene diphosphonate was intravenously injected and data acquisition was initiated approximately 4 h later. SPECT acquisition was performed after planar acquisition. The injected dose, calculated by subtracting the post-injection remaining radioactivity in the tube and syringe from the pre-injection radioactivity, was 952 ± 137 MBq. The uptake time for SPECT acquisition was 250.3 ± 7.9 min.

#### Image acquisition and reconstruction

The SPECT/CT scanner, SPECT acquisition conditions, and CT imaging conditions were the same as for the phantom study. Immediately after data acquisition, a low-dose CT scan was performed. SPECT images were reconstructed using two sets of parameters: OSEM_jaw_, with reconstruction parameters determined by the results of the phantom study; and OSEM_current_, with a subset of 10, number of iterations of 6, and FWHM of 7.80 mm for the Gaussian filter, which were the image reconstruction conditions determined previously using a spherical phantom [25].

#### Data analysis

SUV_mean_ values of the clinical images were calculated by using GI-BONE software (AZE Co., Ltd., Tokyo, Japan). Volumes of interest were placed in the lesion areas. The average SUV that was 40% or more of SUV_max_ in the volume of interest was defined as SUV_mean_. SUV was calculated by

3 $$\text{SUV} = \frac{\text{ SPECT counts}/\text{CCF}}{\text{Injected activity }/\text{Body weight}}$$

The quality of the SPECT images was evaluated visually by a board-certified nuclear medicine physician, a board-certified radiologist, an oral and maxillofacial surgeon, and a board-certified nuclear medicine radiological technologist. The score was graded from 1 to 5 (1, very poor; 2, poor; 3, moderate; 4, good; and 5, excellent) in terms of detectability of lesions, including the contrast and the sharpness of the lesion area.

#### Statistical analysis

SUVs were compared between clinical stages using the Kruskal–Wallis test, and subsequent post hoc analysis using the Steel–Dwass test. The visual score was acquired by averaging the results of the observers’ scores. The visual scores for two parameters were compared by using Wilcoxon signed rank tests. Differences were considered statistically significant when *p* < 0.05.

## Results

### Phantom study

**Fig. 3:**
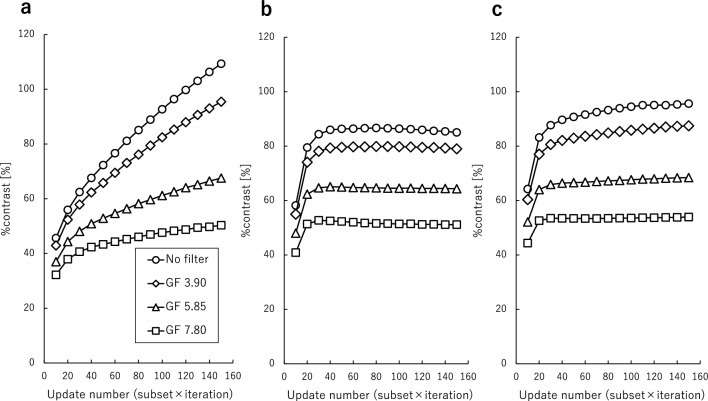
Comparison of the %contrast for LNR of 1:2 (**a**), 1:4 (**b**), and 1:8 (**c**) obtained at various Gaussian filter FWHMs. The number of subsets was set at 10 and the number of iterations varied from 1 to 15. The %contrast increased with update number and converged uniformly, except for LNR of 1:2. *GF* Gaussian filter

**Fig. 4:**
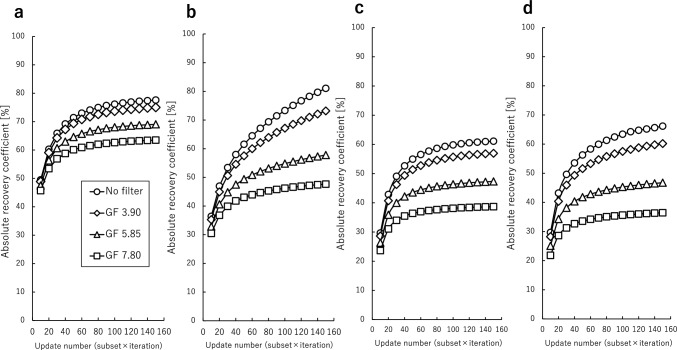
Comparison of the ARC in the normal bone area (**a**), LNR of 1:2 (**b**), 1:4 (**c**), and 1:8 (**d**) obtained at various Gaussian filter FWHMs. The number of subsets was set at 10 and the number of iterations varied from 1 to 15. The ARC increased with update number and converged uniformly, except for LNR of 1:2. *GF* Gaussian filter.

**Fig. 5:**
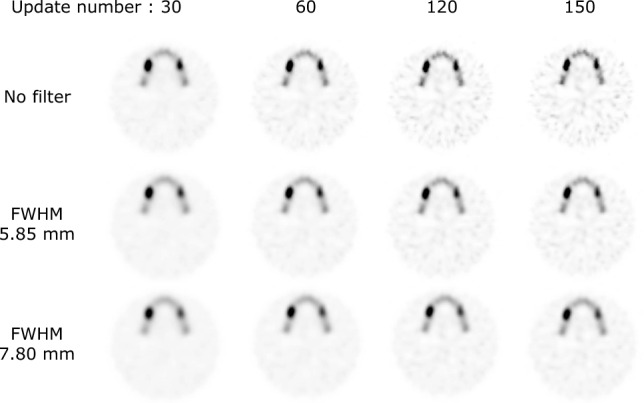
SPECT images of the phantom using various iteration numbers and Gaussian filter FWHMs. The simulated lesions became sharper with increasing iteration number and decreasing Gaussian filter FWHM. The right side of the phantom was LNR 1:8 and the left side was LNR 1:4

The %contrast and ARC plotted as a function of the update number are shown in Figs. 3 and 4 for phantoms with different LNRs. For all LNRs, %contrast and ARC increased with increasing update number or decreasing Gaussian filter FWHM. For %contrast and ARC, the parameters without the Gaussian filter provided the best approximations to the theoretical values. The ARC converged uniformly for both normal bone and lesions with an update number of 120 or larger, except for an LNR of 1:2. In 1:2 LNR, overestimation occurred when the number of updates exceeds 130. The SPECT images obtained with various update numbers and FWHMs of the Gaussian filter are shown in Fig. 5. Visually, although the background uniformity increased with decreasing update number and increasing Gaussian filter FWHM, the clarity of the lesion inside the jawbone decreased. In addition, more than 120 updates were required to obtain sufficient lesion clarity. Based on these results, the optimum parameters determined by the newly developed phantom study (OSEM_jaw_) were 120 updates and no filter.

### Clinical study

Reconstruction parameters for OSEM_jaw_ were 10 subsets, number of iterations of 12, and no filter and those for OSEM_current_ were 10 subsets, number of iterations of 6, and Gaussian filter FWHM of 7.8 mm. OSEM_jaw_ included the image reconstruction parameters determined by the novel phantom, whereas OSEM_current_ included the image reconstruction parameters determined by the spherical phantom in a previous study [25].

**Fig. 6:**
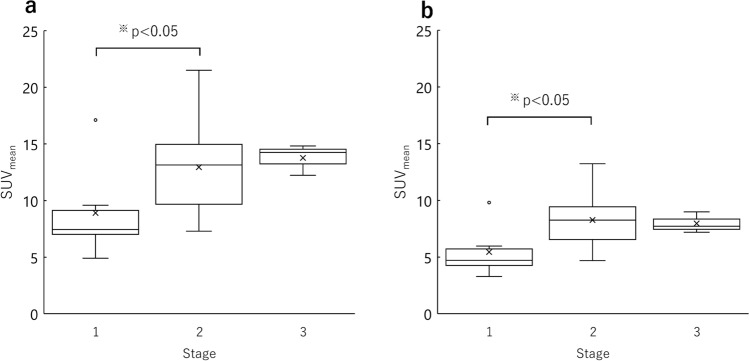
Relationship between SUV_mean_ of the lesion area and clinical stage. SUVs were obtained with OSEM_jaw_ (**a**) and OSEM_current_ (**b**). OSEM_jaw_ includes the image reconstruction parameters determined by the new phantom, whereas OSEM_current_ includes those previously determined by a spherical phantom [25]. Only SUV_mean_ obtained from OSEM_jaw_ increased with disease stage, while OSEM_current_ showed a decrease in mean SUVmean from stage 2 to stage 3. Significant differences were observed only in the parameters across clinical stages (*p* < 0.05). Subsequent post hoc analysis revealed that SUV_mean_ for stage 2 was significantly higher than that for stage 1

SUV_mean_ and clinical stage obtained by OSEM_jaw_ and OSEM_current_ are compared in Fig. 6. The mean and standard deviation of SUV_mean_ obtained from OSEM_jaw_ were 2.3 ± 0.6 for normal area, 8.9 ± 1.4 for stage 1 lesions, 12.9 ± 4.1 for stage 2 lesions, and 13.8 ± 1.4 for stage 3 lesions. For OSEM_current_, they were 1.4 ± 0.4 for normal area, 5.4 ± 2.3 for stage 1 lesions, 8.3 ± 2.5 for stage 2 lesions, and 8.0 ± 0.9 for stage 3 lesions. Only SUV_mean_ obtained from OSEM_jaw_ increased with increasing disease stage, while OSEM_current_ showed a decrease in mean SUVmean from stage 2 to stage 3. Significant differences were observed in both parameters across clinical stages. Subsequent post hoc analysis revealed that SUV_mean_ for stage 2 was significantly higher than that for stage 1.

**Fig. 7:**
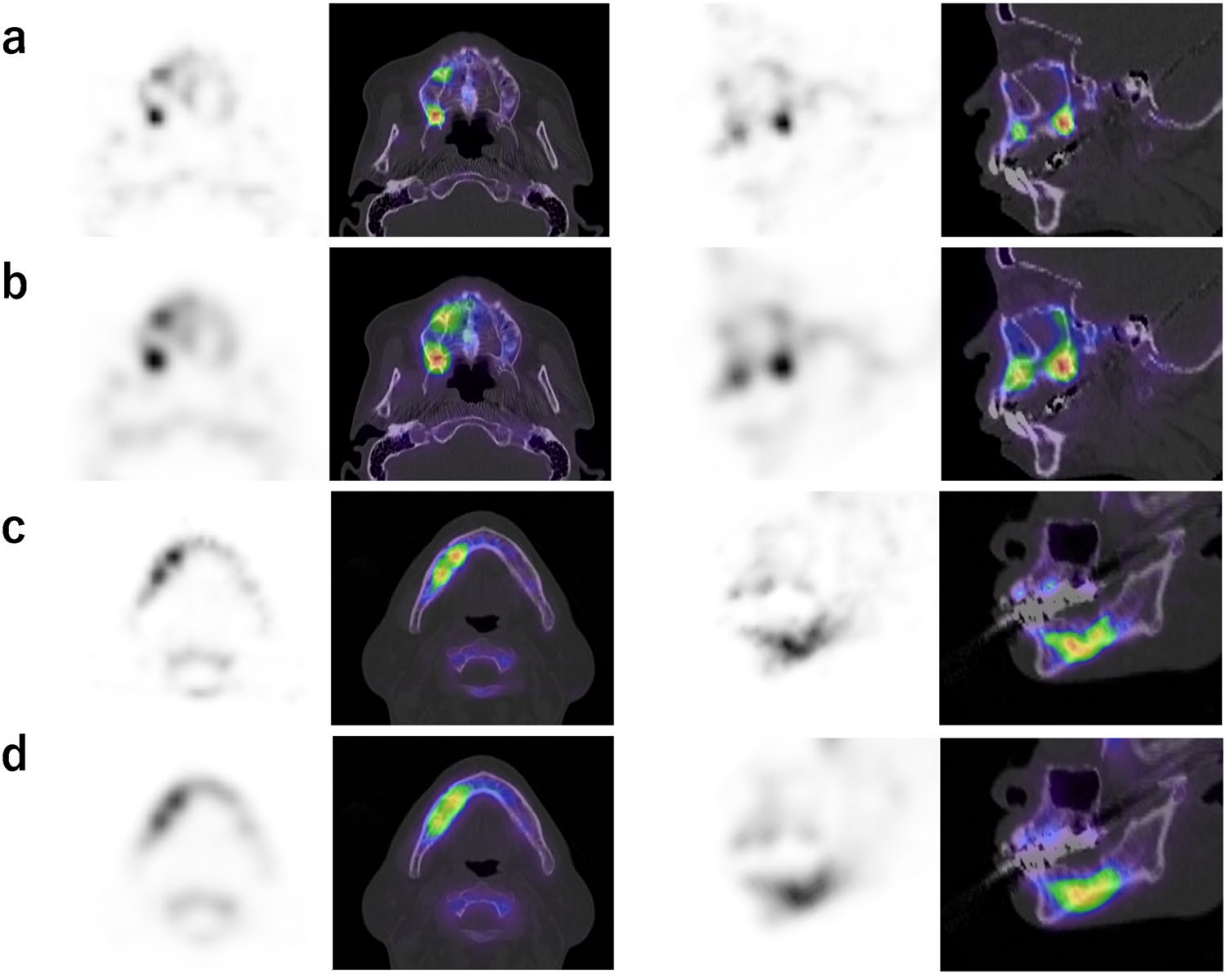
Jawbone SPECT images and fused SPECT/CT images of MRONJ of the maxilla (case 4) and mandible (case 10) using OSEM_jaw_ (**a**, **c**), OSEM_current_ (**b**, **d**). In case 4, OSEM_jaw_ clearly improved the contrast and sharpness of the lesion area. The slice presented a transaxial image along the maxilla and a sagittal image along the right maxilla. The SUV_mean_ of the lesion area was 14.8 and 7.2, respectively, and the mean visual score was 4.25 and 3.00. In case 10, OSEM_jaw_ not only clearly distinguished the lesion but also delineated the shadows within the hot area. The SUV_mean_ of the lesion area was 8.1 and 6.9, respectively, and the mean visual score was 3.75 and 3.00

The visually assessed quality of the SPECT images reconstructed by OSEM_jaw_ (3.7 ± 0.9) was significantly superior to that reconstructed by OSEM_current_ (2.9 ± 1.1). Representative SPECT images reconstructed by OSEM_jaw_ and OSEM_current_ are presented in Fig. 7. OSEM_jaw_ clearly improved the contrast and the sharpness of the lesion area. This parameter showed a tendency to achieve higher visual scores, particularly in cases with smaller lesions or maxillary MRONJ.

## Discussion

In this study, the usefulness of a novel phantom developed for evaluating jawbone SPECT examinations was verified via a technical evaluation. In the technical evaluation, optimal image reconstruction conditions for jawbone SPECT were investigated in terms of image quality and quantitative accuracy. The clinical evaluation validated the reconstruction conditions, thus demonstrating the usefulness of this phantom. The phantom was designed based on 3D skull data. The jawbone section had a horseshoe shape to mimic the mandible because about 77.5% of MRONJ occurs in the mandible [26]. The structure of the jawbone section consisted of eight lesion areas set in the normal bone part, allowing for the installation of lesion areas in various locations, because MRONJ occurs throughout the jaw. This phantom has the potential to simulate various oral surgical disease conditions in bone SPECT/CT by changing the concentration of ^99m^Tc and bone-equivalent solution. In this study, the phantom was created by varying only the LNR of the ^99m^Tc solution.

In the phantom study, %contrast and ARC were used as evaluation indices to assess optimal image reconstruction parameters in terms of image quality and quantitative accuracy. The %contrast and ARC increased with the update number and converged uniformly for LNR of 1:4 and 1:8. The convergence of %contrast required 40 updates, whereas the convergence of ARC for each normal bone and lesion required 120 updates. The %contrast and ARC did not converge at LNR of 1:2, overestimation occurred when the number of updates exceeds 130. Based on these results, 120 updates was used as the optimal update number. At LNR of 1:4, %contrast and ARC varied by up to 28.5% and 31.5%, respectively, with changes in the update number. As the Gaussian filter FWHM increased, %contrast and ARC decreased. At LNR of 1:4, %contrast and ARC varied by up to 35.1% and 22.4%, respectively, with changes in the Gaussian filter FWHM. These results demonstrate that both the update number and the Gaussian filter FWHM are important factors in the assessment of the quantitative accuracy and image quality. The optimal reconstruction condition depends on the size and physical properties of the target. Jawbone SPECT required conditions less susceptible to partial volume effects because the jawbone is thin and lesions occur within it. Compared to previous studies, the proposed condition includes a large number of updates, and no facility specifically implements the non-use of filters [3–7]. In terms of quantitative accuracy, the parameters used in previous studies may have been inadequate. Although Gaussian filters are used to improve background uniformity, jawbone SPECT has a high contrast with the background. In this study, the ratio of ^99m^Tc solution inclusion concentration of normal bone to background was 13:1 based on clinical data. For high-contrast objects, background uniformity is not considered important. Decreased background uniformity leads to false positives and false negatives, although this effect diminishes when there is a large contrast with the target. In the visual assessment, no such cases were observed. Therefore, in terms of image quality and quantitative accuracy, we considered it most appropriate to use no filter. In addition, the protocol in this study set a sufficient dose of radioactivity and acquisition time compared to previous studies [3–7].

The clinical evaluation validated the image reconstruction parameters determined by the new phantom (OSEM_jaw_). In the validation, we used the image reconstruction parameters previously determined by a spherical phantom (OSEM_current_) for comparison [25]. Images generated from the two image reconstruction parameters were used to evaluate the correlation between SUV_mean_ and clinical staging, and visual evaluation. OSEM_jaw_ showed higher correlation between SUV_mean_ and clinical staging compared with OSEM_current_. The high spatial resolution parameters established in the phantom study showed significant results in the clinical evaluation. One noteworthy finding was that stage 3 had a higher mean SUV_mean_ than stage 2 in OSEM_jaw_ only. Stage 3 included cases with small accumulations due to the presence of necrotic areas inside the lesion (Fig. 7, case 4). Small accumulations are prone to being measured at lower SUVs than their actual levels due to the significant influence of partial volume effects. The high spatial resolution of OSEM_jaw_ may have enabled it to measure SUV_mean_ with greater accuracy, reflecting the high inflammatory activity. In MRONJ, accurate assessment of inflammatory activity is crucial for monitoring of inflammatory activity and determining the extent of surgical resection [1–3, 27]. The highly accurate SUVs obtained from OSEM_jaw_ may contribute not only to stage classification but also to these purposes.

In addition, OSEM_jaw_ tended to visual score higher than OSEM_current_, particularly in cases with small lesions and in maxillary MRONJ cases. In case 4, a cold-in-hot pattern was observed due to necrosis within the MRONJ lesion. OSEM_jaw_ clearly highlighted the small areas of accumulation and the cold regions, while also depicting the bony structure of the maxilla with clarity in the normal bone. Similarly, in case 10, the lesion was not only distinctly defined, but the shading within the hot area was also distinguishable. Miyashita et al. stated that residual sites of high activity after surgical therapy can induce recurrence [3], and OSEM_jaw_ may have the potential to accurately identify these areas.

However, the novel phantom was designed with a structure mimicking the mandible. The anatomical structure, cortical bone thickness, and marrow characteristics differ significantly between the maxilla and mandible. The phantom consisted of eight lesion regions set in the normal bone portion, but the lesions were placed only in the same region on the left and right sides. These regional differences may affect the optimal parameters, necessitating further validation.

We concluded that the major factor in the superior results obtained with OSEM_jaw_ was the design of the novel phantom to resemble the shape of a human jawbone and accurately reflect the attenuation coefficient. Changes in attenuation coefficient affect image quality and quantitative accuracy [23]. These results indicate that the optimal parameter settings using the jaw phantom contribute to the improved image quality and quantitative accuracy in clinical examination.

Our study has several limitations. First, the phantom study was conducted by varying only the LNR of the 99mTc solution. In MRONJ, not only the LNR but also the accumulation volume changes as the disease progresses. In addition, osteoblastic and osteolytic changes occur in CT image findings.

In this study, the attenuation coefficient in the bone region was kept constant, but an attenuation coefficient that reflects these findings must be set. Second, we did not evaluate the reproducibility of the radioactivity concentrations in the SPECT image. Further studies are required to evaluate these points. Third, we examined only one commercially available SPECT/CT device and iterative reconstruction technologies, namely, Flash 3D (Siemens AG). Therefore, a multicenter study is required to investigate and verify the usefulness of the phantom in technical studies. Although this point is a limitation in all jawbone phantom studies, phantom studies are important for optimization, standardization, and harmonization of SPECT images and we believe that our phantom may play an important role in jawbone SPECT.

## Conclusion

We developed a novel phantom suitable for technical evaluation of bone SPECT targeting medication-related osteonecrosis of the jaw. The phantom was used to determine the optimal reconstruction parameters for jawbone SPECT and verified the validity of the parameters through clinical study. This study demonstrated the usefulness of the newly developed phantom.
